# Sword-Like Trauma to the Shoulder with Open Head-Splitting Fracture of the Head

**DOI:** 10.1155/2016/3539503

**Published:** 2016-07-05

**Authors:** Andreas Panagopoulos, Konstantinos Pantazis, Ilias Iliopoulos, Ioannis Seferlis, Zinon Kokkalis

**Affiliations:** Department of Shoulder & Elbow Surgery, Patras University Hospital, Papanikolaou 1, 26504 Patras, Greece

## Abstract

Head-splitting fractures occur as a result of violent compression of the head against the glenoid; the head splits and the tuberosities may remain attached to the fragments or split and separate. Isolated humeral head-splitting fractures are rare injuries. Favorable results with osteosynthesis can be difficult to achieve because of the very proximal location of the head fracture and associated poor vascularity. We present a case of a 67-year-old man who sustained a severe, sword-like trauma to his left shoulder after a road traffic accident with associated isolated open Gustilo-Anderson IIIA humeral head-splitting fracture. Bony union was achieved with minimal internal fixation but the clinical outcome deteriorated due to accompanying axillary nerve apraxia. To our knowledge, this type of sword-like injury with associated humeral head-split fracture has not previously been reported.

## 1. Introduction

Head-splitting fractures are extremely rare and indicate a severe trauma to the shoulder joint. The head is violently compressed against the glenoid and split with or without associated dislocation. We present a case of an isolated open Gustilo-Anderson IIIA humeral head-splitting fracture after a road traffic accident treated with minimal internal fixation. To our knowledge, this type of sword-like injury has not previously been reported.

## 2. Case Presentation

**Figure 1:**
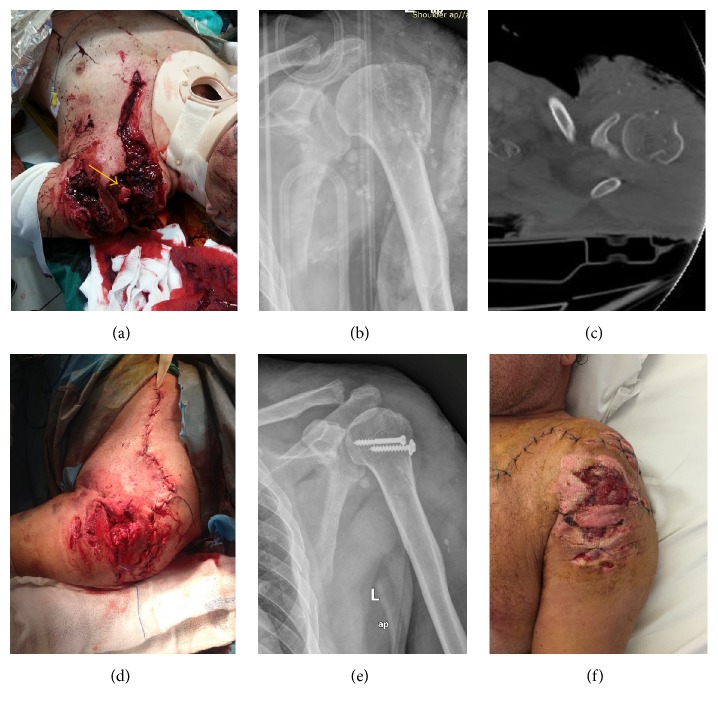
(a) Photography of the trauma during patient resuscitation indicated severe, sword-like injury to the left shoulder, with open fracture of the humeral head (arrow). (b) Preoperative anteroposterior X-ray of the left shoulder showing head-splitting fracture of the proximal humerus and presence of multiple foreign bodies (glass). (c) CT scan of the left shoulder indicating involvement of ~30% of the articular surface and the greater tuberosity. (d) Intraoperative picture after muscle and skin closure. (e) Postoperative X-ray of the left shoulder showing adequate reduction of the fragment. (f) Condition of the skin at the 10th postoperative day just before a split skin graft was about to apply.

A 67-year-old man was admitted to our department following a high-speed road traffic accident. He was sited at the back of a car when a lateral-frontal collision happened crushing the back door to his left shoulder. He sustained an open, Gustilo-Anderson IIIA fracture of the proximal humerus with a large overlying sword-like contaminated wound and significant skin loss (Figure 1(a)). There was no neurovascular deficit to the distal forearm and hand. He sustained also fractures of the 1st, 2nd, and 6th rib without any intrathoracic injury. Plain radiographs and CT scan indicated an anteroposterior directed head-splitting fracture of the humeral head involving ~30% of the lateral articular surface with a sagittal extension pattern of the greater tuberosity and without any evidence of humeral head dislocation (Figures 1(b) and 1(c)). He was transferred immediately to the operating theatre as there was uncontrolled bleeding from the wound and hemoglobin level had been dropped by 4 degrees during resuscitation.

At surgery, thorough debridement was carried out and foreign bodies (glass particles) were removed where possible. There was a large circumferential wound to the deltoid involving its anterior, middle, and part of its posterior fibers. The platysma muscle was also ruptured exposing the medial and lateral end of the clavicle. The fractured head was palpable under the deltoid being totally uncovered from rotator cuff muscles. Severe bleeding was detected from the posterior circumflex artery and vein; both vessels were ligated. The axillary artery and the brachial plexus were recognized unmarked. We were not able to identify the axillary nerve at that time. The fractured humeral head was reduced with pointed clamps and fixed with two 4 mm AO cancellous screws (Figure 1(e)). The torn RC was repaired primarily with transosseous nonabsorbable sutures. Deep soft tissue closure was achieved by loosely approximated absorbable sutures (Figure 1(d)). Skin has been closed partially and a Penrose drain was applied. From the wound was cultivated* E. coli* and* Staphylococcus warneri*. The patient was treated with intravenous antibiotics for 3 weeks and oral administration for another 3 weeks after his discharge. At the 10th postoperative day, the wound showed signs of reepithelialization and a split-thickness skin graft was applied for terminal closure (Figure 1(f)).

**Figure 2:**
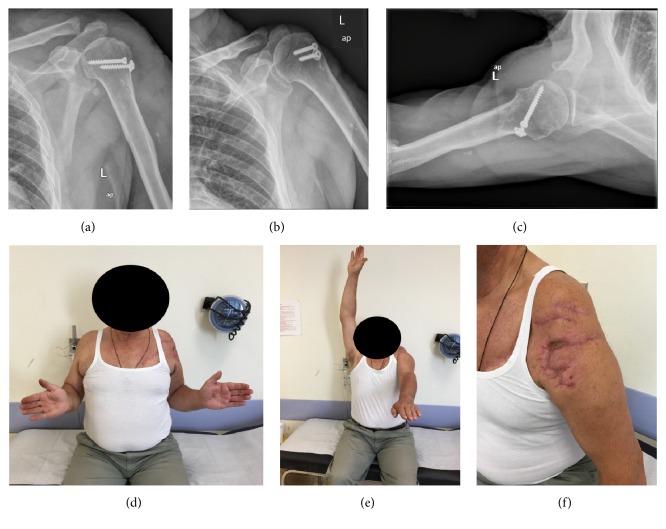
((a)–(c)) Radiological examination at 12 months with anteroposterior views in external (a) and internal (b) rotation as well as axillary view of the shoulder (c) indicated solid union of the fracture. ((d)-(e)) Poor clinical outcome especially in forward elevation due to axillary nerve neurapraxia. (f) Clinical picture of the wound at 17 months after surgery.

The arm was placed in a sling for 4 weeks. Shoulder physiotherapy and passive assisted mobilization were commenced as soon as the wound was closed, at the second postoperative week. Soft tissue and bony healing occurred without further surgical intervention. The humeral head fracture united with no evidence of avascular necrosis, confirmed radiologically within 12 months (Figures 2(a)–2(c)). The patient unfortunately did not recover shoulder abduction and forward elevation as an ENG assessed a complete neurapraxia of the axillary nerve (Figures 2(d)–2(f)). He had a Constant score of 47 at the latest clinical follow-up 17 months postoperatively. A nerve transfer has been offered to him but he denied any further surgical intervention.

## 3. Discussion

Head-splitting fractures indicate a severe trauma to the shoulder joint. The head is violently compressed against the glenoid and split. A segment of the humeral head is fractured and is subluxated or dislocated, while the articular surface of the unfractured part of the head remains attached to the shaft. Neer II [1] defined splitting fractures as those in which the fractured fragments measure more than 20% of the articular surface. Classic radiographic “trauma series” of the shoulder and computed tomography are valuable for delineating the configuration of the fracture and helping to plan surgical reconstruction [2, 3]. Robinson et al. [4] proposed two patterns of injury in complex humeral head fractures with dislocation (or splitting) depending on a prospective assessment of the pattern of soft tissue and bony injury and the degree of devascularisation of the humeral head. In type I injuries, the head retains capsular attachments and arterial back-bleeding whereas in type II injuries the head is devoid of significant soft tissue attachments with no active arterial bleeding. ORIF is recommended in type I injuries as only two of 23 patients with type I injuries developed radiological evidence of osteonecrosis of the humeral head, compared with four of seven patients with type II injuries. The mechanism of injury in our case was more unique as the split in the humeral head was probably caused by direct extrinsic trauma to the shoulder by the distorted metallic parts of the back door during the crush. This explains the severe damage to deltoid and rotator cuff muscles as well as the neurologic damage to the axillary nerve.

The outcome of head-split fractures, regardless of management, is thought to be worse than other types of humeral head fractures because of a perceived higher energy of injury and disruption of the terminal blood supply to the articular fragments [5]. Lee and Hansen [6] reported on 19 patients with displaced 4-part fracture or fracture dislocation treated with ORIF and having no signs of AVN after a mean follow-up period of 23.6 months. They hypothesized a mechanism of revascularization with capillary ingrowth sequence and new bone formation during the healing process (e.g., creeping substitution). As the humeral head is surrounded by rich vascular tissue and has wide fractured surfaces relative to the thickness of the head, a reduced mechanical stress is expected as the healing is progressed, thus reducing the incidence of AVN. The available evidence on optimal treatment of head-splitting fractures is scarce: apart from some case reports [7–9], only two case series have been published on minimal [10] or locking plate osteosynthesis [11], one case series has been published on hemiarthroplasty [12], and two recent studies have been published on reverse shoulder arthroplasty [13, 14] of isolated head-splitting fractures.

Collopy and Skirving [7] reported on a 20-year-old patient who sustained a “transchondral fracture dislocation” involving 60% of the articular surface and fixed with two 4.0 mm cancellous screws; at 7-year follow-up, the patient had full range of motion and no evidence of AVN or arthritis. Gokkus et al. [8] reported a complex head-splitting fracture with anterior dislocation of the fractured part on a 40-year-old patient. Surgery was performed within 6 hours and the osteochondral fragment, carrying approximately 65% of the articular surface, was found firmly entrapped between the anterior glenoid rim and the subscapularis. Anatomical reduction was achieved with two k-wires and three 4 mm AO cancellous screws. After the 15-month follow-up, the patient had 130° of forward elevation, no shoulder pain, and a Constant score of 76 points. Bailie and McAlinden [9] reported a case of a 17-year-old man with a compound comminuted fracture of the proximal third of the humeral shaft with complete head-splitting extension and a large overlying contaminated wound with skin loss (Gustilo-Anderson grade IIIB). This is the only case in the literature describing an open head-splitting fracture. The mechanism of injury was high-speed road traffic accident. At surgery, fixation with a bridging plate was impossible as the proximal third of the humeral shaft was found to be highly comminuted with marked degloving and soft tissue stripping of multiple fragments in this segment. Fixation of the head part was achieved with two fully threaded AO cancellous screws. The humeral shaft fracture united fully both clinically and radiologically within 3 months without the need for further surgery or bone grafting. At the final follow-up, 8 months after surgery, the patient recovered shoulder abduction and flexion >130°.

Chesser et al. [10] reported on 8 patients with head-splitting fractures; five were diagnosed at presentation and treated with minimal fixation (3 cases), primary hemiarthroplasty (1 case), and closed reduction (1 case). In three cases, the injury was initially unrecognized; two developed a painless bony ankyloses and one was scheduled for hemiarthroplasty. At the latest follow-up (minimum of two years), all ORIF patients had no signs of AVN of the humeral head. Gavaskar and Tummala [11] reported the largest study so far including 15 patients <55 years old treated with locking plate fixation. Five fractures were classified as simple (isolated head-splitting fractures) and 11 as complex (associated tuberosity fractures). None of the patients with simple fracture developed AVN; a nonunion rate of 20% and AVN rate of 40% in complex fractures indicated the inherent severity of these injuries. The mean Constant (66.5) and DASH score (21) showed significantly better outcomes in simple fractures. Greiwe et al. [12] reported the outcome of primary hemiarthroplasty in 8 head-splitting fractures in contrast to 22 patients with complex 3- and 4-part fractures. Head-split fractures demonstrated improved range of motion (mean 138°), complication rate (12.5%), and revision rate (0) compared with standard fractures at an average of 3.6 years postoperatively. Finally, primary reverse shoulder arthroplasty for isolated head-splitting fractures or split fractures with long segment diaphyseal extension is another treatment option in older patients (>65 years old) as indicated by two recent reports describing mixed population of complex fractures of the proximal humerus [13, 14].

## 4. Conclusion

In our case, the mechanism of injury was direct open trauma not previously reported. A head preserving treatment was considered within few hours of the injury. The humeral head was reduced and fixed with minimal internal fixation and soft tissue handling paying attention to avoiding excessive soft tissue stripping. Clinical and radiological healing was uncomplicated. Final outcome was poor due to axillary nerve apraxia but the patient refused any further intervention. There is little advice about the optimal treatment of splitting fractures of the humeral head in the literature. Internal fixation is mandatory for younger patients but the results are worse in more complex fracture patterns, including fractures of tuberosities. Hemiarthroplasty or reverse arthroplasty can be used in older, low demanded patients.
